# Supplementary material for: Efficient fluorescence‐based localization technique for real‐time tracking endophytes route in host‐plants colonization
Source: Plant Direct. 2022 Aug 8;6(8):e427. doi: 10.1002/pld3.427 (PMC9360559; doi:10.1002/pld3.427)
Supplement: Supplementary file 1 — Figure S1. Effects of the freezing on competent and transformed BCA's growth. a, Growth characteristics of competent BCAs with control non‐treated (CTR, white columns graph), CaCl2‐treated (gray columns graph), and TRIS‐treated (black columns graph) BCAs. b, Growth characteristics of transformed competent BCAs with control non‐transformed competent CaCl2‐treated (CTR‐CaCl2 white columns graph), transformed competent CaCl2‐treated (gray bars graph), control non‐transformed competent TRIS‐treated (CTR‐TRIS, light gray bars graph), and transformed competent TRIS‐treated (black bars graph) BCAs. The data depicted in the graphs correspond to the mean ± SD of two replications of culture events (n = 3 plates scored per culture event for each BCA strain). Table S1. Transformation efficiencies of the pANIC‐10A‐mediated transformation of BCA cells. The numbers represent the efficiencies of the plasmid pANIC‐10A transformation of BCA strain IMC8, PRT, PS, and PSL cultures. The CaCl2‐ or TRIS‐induced competent BCA cells were transformed simultaneously at the final concentration of inoculum OD600 = .5. The transformation efficiency was evaluated by scoring a growing kanamycin‐resistant (KAN R ) colony‐forming unit (CFU). The data depicted in the table corresponds to the mean ± SD of two replications of transformation events (n = 3 plates scored per transformation event for each BCA strain). Figure S2. Double‐localization of PSL‐pBSU101/eGFP and IMC8‐pANIC‐10A/pporRFP populations within 7‐d‐old co‐colonized sorghum Topper 76–6 root. Microscopy micrographs of fluorescent PSL‐pBSU101/eGFP and IMC8‐pANIC‐10A/pporRFP populations in sorghum Topper 76–6 roots. A and a1, bright‐field, b and b1, Green fluorescent PSL‐pBSU101/eGFP population c and c1, Red fluorescent IMC8‐pANIC‐10A/pporRFP population. B1‐c1 and b2‐c2, overlapped micrographs showing green PSL‐pBSU101/eGFP and red IMC8‐pANIC‐10A/pporRFP populations in the same roots. Square frames represent the area of magnified pictures a1 [file PLD3-6-e427-s001.pdf]

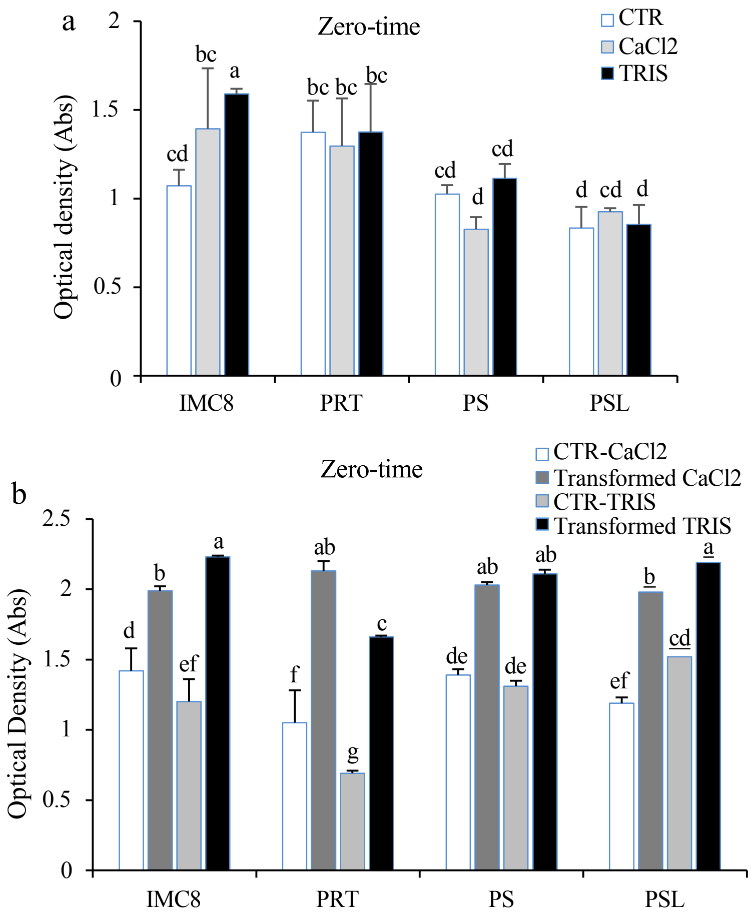

Supplementary Figure 1. Effects of the freezing on competent and transformed BCA's growth. **a**, Growth characteristics of competent BCAs with control non-treated (CTR, white columns graph), $CaCl_2$-treated (gray columns graph), and TRIS-treated (black columns graph) BCAs. **b**, Growth characteristics of transformed competent BCAs with control non-transformed competent $CaCl_2$-treated (CTR-$CaCl_2$ white columns graph), transformed competent $CaCl_2$-treated (gray bars graph), control non-transformed competent TRIS-treated (CTR-TRIS, light gray bars graph), and transformed competent TRIS-treated (black bars graph) BCAs. The data depicted in the graphs correspond to the mean ± SD of two replications of culture events (n = 3 plates scored per culture event for each BCA strain).

		pANIC-10A					
			$CaCl_2$		TRIS		
BCA strain	Selection antibiotic	Total no. of CFU grown	Total no. of Resistant CFU	Percentage efficiency (%)	Total no. of CFU grown	Total no. of Resistant CFU	Perce[illegible] effici[illegible] (%[illegible]
IMC8	*KAN*^{R}	198±44.4	2±1	0.99±0.37c	1289±119.15	759.67±94.88	59.46±1[illegible]
PRT	*KAN*^{R}	126.33±3.21	1.67±0.58	1.32±0.45c	790.33±60.08	526±99.02	66.82±1[illegible]
PS	*KAN*^{R}	86±11.27	2.67±0.58	3.11±0.65c	886.33±51.6	573±44.31	64.6±1.4[illegible]
PSL	*KAN*^{R}	32±8.72	1.33±0.58	4.06±0.62c	1017.33±112.15	763±51.42	75.37±6.[illegible]

Supplementary Table 1. Transformation efficiencies of the pANIC-10A-mediated transformation of BCA cells.

The numbers represent the efficiencies of the plasmid pANIC-10A transformation of BCA strain IMC8, PRT, PS, and PSL cultures. The $CaCl_2$- or TRIS-induced competent BCA cells were transformed simultaneously at the final concentration of inoculum $OD_{600} = 0.5$. The transformation efficiency was evaluated by scoring a growing kanamycin-resistant (*KAN*^{R}) colony-forming unit (CFU). The data depicted in the table corresponds to the mean ± SD of two replications of transformation events (n = 3 plates scored per transformation event for each BCA strain).

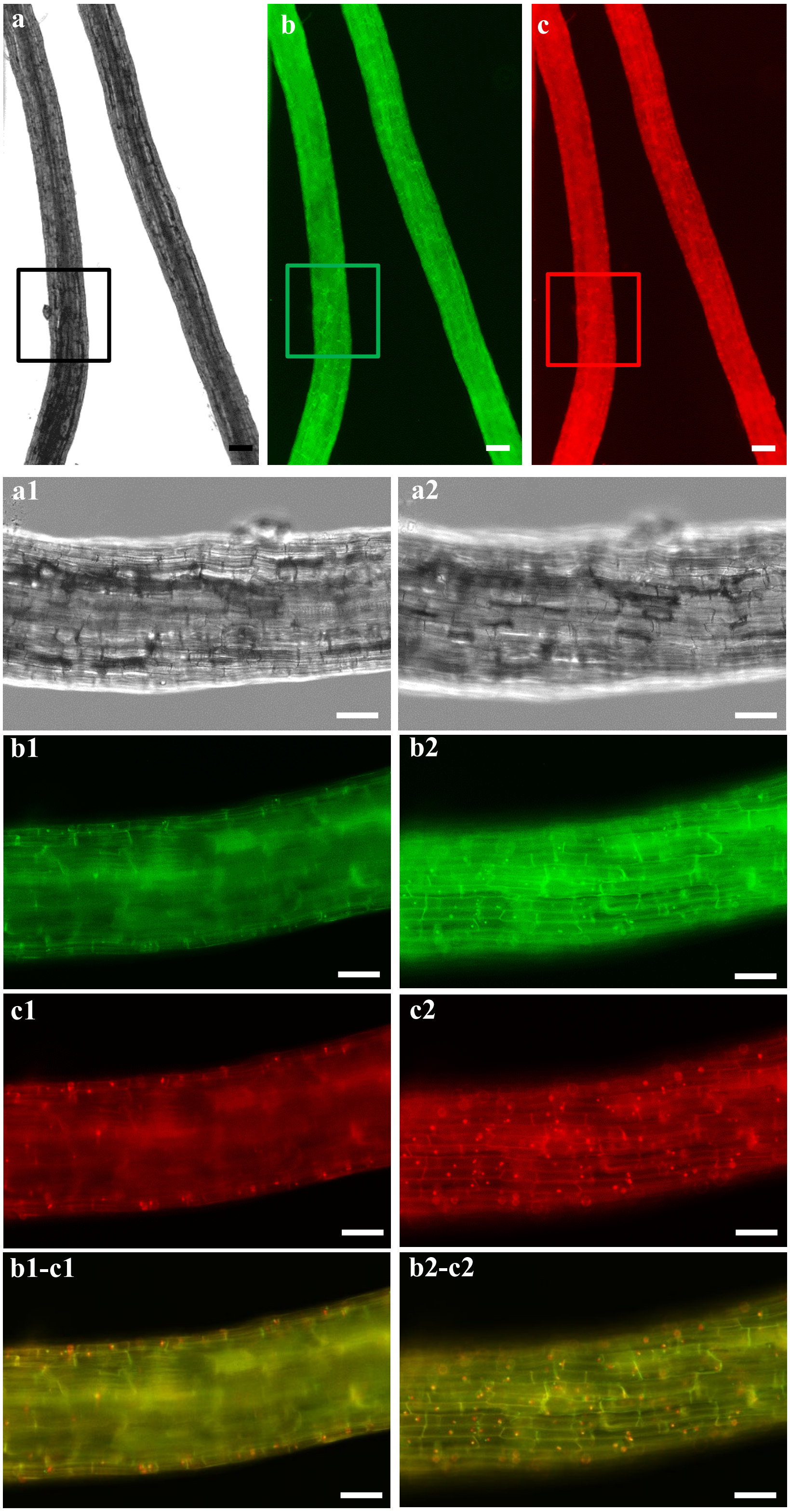

Supplementary Figure 2. Double-localization of PSL-pBSU101/eGFP and IMC8-pANIC-10A/pporRFP populations within 7-d-old co-colonized sorghum Topper 76-6 root.

Microscopy micrographs of fluorescent PSL-pBSU101/eGFP and IMC8-pANIC-10A/pporRFP populations in sorghum Topper 76-6 roots. **A** and **a1**, bright-field, **b** and **b1**, Green fluorescent PSL-pBSU101/eGFP population **c** and **c1**, Red fluorescent IMC8-pANIC-10A/pporRFP population. **B1-c1 and b2-c2**, overlapped micrographs showing green PSL-pBSU101/eGFP and red IMC8-pANIC-10A/pporRFP populations in the same roots. . Bars = 100 µm **a** to **c**., 50 µm **a1** to **b2-c2**.

Supplementary Table 2. Selected BCA strains characteristics.

Strains	Source/Description	Functions	Reference
Bacillus thuringiensis (IMC8)	Flowering dogwood branch	Control of Dogwood	Rotich et al. 2015
Bacillus subtilis (PRT)	Papaya stem	Control of Phytophthora root rot	Mmbaga et al. 2018a
Bacillus vallismortis (PS)	Papaya stem	Control of Phytophthora root rot	Mmbaga et al. 2018a
Bacillus amyloliquefaciens (PSL)	Papaya stem	Control of Macrophomina root rot	Joshua et al. 2017<br>Mmbaga et al. 2018b

Supplementary Table 3. Plasmids and sequences of *HYG*, *eGFP,* and *pporRFP* gene primers used for PCR.

Plasmids		Description	References
pBSU101		*SPEC*^{r}, carrying *eGFP* under the control of the *cfb* promoter of *Streptococcus agalactae*	Aymanns et al. 2011
pANIC-10A		*KAN*^{r}, *HYG*^{r}, carrying *pporRFP* under the control of the *cfb* promoter of *Streptococcus agalactae*	Mann et al. 2012
Gene name	Gene unitranscript ID	Primers	
HYG	MG725339	FP: 5'-ATGAAAAAGCCTGAACTCACCGCGAC-3'<br>RP: 5'-CTATTTCTTTGCCCTCGGACGAGTGC-3'	Wuddineh et al. 2015<br>Ondzighi-Assoume et al. 2019
eGFP	AAB02572	FP: 5'-CCT GAA GTT CAT CTG CAC CA-3'<br>RP: 5'-GGT CTT GTA GTT GCC GTC GT-3'	Ondzighi-Assoume's lab primers designed
pporRFP	DQ206380	FP: 5'-TTT CAA AGC AAA GTG GGG TC-3'<br>RP: 5'-CAC CAT CTT CAA AGG TCA TG-3'	Mann et al. 2012a<br>Mann et al. 2012b<br>Ondzighi-Assoume et al. 2019
